# Validity of self-reported out-of-school physical activity among Finnish 11-year-old children

**DOI:** 10.1186/s13690-016-0123-2

**Published:** 2016-02-08

**Authors:** Suvi Määttä, Teija Nuutinen, Carola Ray, Johan G. Eriksson, Elisabete Weiderpass, Eva Roos

**Affiliations:** Samfundet Folkhälsan, Folkhälsan Research Center, Helsinki, Finland; Department of Public Health, University of Helsinki, Helsinki, Finland; Department of Community Medicine, Faculty of Health Sciences, UiT The Arctic University of Norway, Tromsø, Norway; Department of Research, Cancer Registry of Norway, Oslo, Norway; Department of Medical Epidemiology and Biostatistics, Karolinska Institutet, Stockholm, Sweden; Department of Chronic Disease Prevention, National Institute for Health and Welfare, Helsinki, Finland; Department of General Practice and Primary Health Care, University of Helsinki, Helsinki, Finland; Vasa Central Hospital, Vasa, Finland; Unit of General Practice, Helsinki University Central Hospital, Helsinki, Finland

**Keywords:** validation studies, health behavior, physical activity, exercise, assessment tool

## Abstract

**Background:**

The aim of this study is to assess the repeatability and validity of the Finnish 11-year old children’s out-of-school physical activity (PA) questionnaire using accelerometer as reference method. A sub-sample of children (*N* = 155, 60 % participant rate) participating in the Finnish Health in Teens study was recruited in 2013. Children completed a questionnaire measuring PA two times, and wore an accelerometer for seven days. The questions and accelerometer data were transformed into average minutes of behaviors per day. Repeatability was measured by intra-class correlations. To test validity, Spearman correlations between the questions and accelerometer was checked and the Bland-Altman model was conducted. Kruskall-Wallis tests were conducted to examine the ranking capability of questionnaire.

**Results:**

The intra-class correlations between two measurement times of questionnaire had substantial agreement. The Spearman correlations between the questions and accelerometer were poor. Based on Kruskal-Wallis tests, the questionnaire was moderately able to rank children according to their levels of PA.

**Conclusions:**

The repeatability of the questionnaire had substantial agreement among 11-years-old, whereas it moderately classifies objectively measured PA. If the aim is to measure children’s duration of PA, the accelerometer might be a better measurement method to use among 11-year old children. If the aim is to classify children according to their behavior, then the used questionnaire is moderately appropriate.

## Background

Children’s frequent and regular physical activity (PA) is an important part of a healthy lifestyle in order to prevent obesity-related and chronic diseases [1, 2]. Accurate assessment for PA among children is therefore a public health importance, especially in out-of-school-hours, because the PA after school hours is low [3]. In Finland, the out-of-school hours is especially interesting for the age group of 11-year old children, because the overall PA levels begin gradually decrease at this age [4]. Secondly, 11-year old Finnish children are usually 5 hours per day in the school [5], and the afternoon clubs in school, such as PA clubs, are uncommon [6]. Thus, the PA variation occurs mainly during out-of-school hours. A validated questionnaire that is able to discriminate between physically active and inactive in out-of-school hours is therefore useful.

However, forming a validated questionnaire among children has been difficult. According to recent review [7, 8], the repeatability of PA questionnaires among children varies usually between acceptable to good, and the validity of PA questionnaires varies between poor to moderate [7]. A reason for this poorer recall might be that PA is accumulated throughout the day and the number and diversity of PA is great [9–11]. However, focusing on certain periods of day such as out-of-school-hours in a questionnaire might be an easier task for children [12]. The questionnaires should be validated against objective measurement before their use, because questionnaires are prone to recall biases and to social desirability leading to misreporting of behaviors [13, 14]. Different objective measurements, such as doubly labeled water, heart rate monitoring or accelerometers are used as golden standards for validating questionnaire [7]. To free-living children, the accelerometer is considered less-burden compared to other methods [15] and the separation of out-of-school-hours from other time points is possible.

The aim of this research is to develop a reliable and valid questionnaire to measure out-of-school PA. Our specific aims were: 1) to test the re-test repeatability of children’s out-of-school PA questionnaire; 2) to test the questionnaire’s validity using an accelerometer as an objective measure and 3) to test if the questionnaire can correctly rank the 11-year old children according to the objectively measured PA in out-of-school-hours.

## Methods

### Study design

A convenience sample of children (*N* = 282) who were participating in the Finnish Health in Teens survey (Fin-HIT) was recruited during spring 2013. In total, 17 schools were contacted and of these schools, 12 schools participated in this sub-study.

After schools’ willingness to participate, the parent and the child gave their informed consent to participate in this sub-study. The introductions of using accelerometer and completing diary for the participating children were given in a school lesson and in written form. The study was approved by the Coordinating Ethics Committee of the Helsinki and Uusimaa Hospital District.

### Questionnaire

The participating children completed an out-of-school PA questionnaire twice (approximately 30 days apart). Two out-of-school PA questions were asked. The out-of-school PA was defined as moderate-to-vigorous PA (MVPA) that child was doing alone, in sport clubs, and with family or friends. PA in schools and during school trips were not asked to take into account. In the first question, children were asked to evaluate how many hours per week they were physically active in out-of-school-hours (hours/per week). 10 response options were possible, ranging from ‘one hour or less per week’ to ‘ten hours or more per week’. For the analyses, two types of variables were formed: a) the weekly MVPA was transformed into minutes and divided by seven to generate the average daily activity time (min/day) called as MVPA Duration (continuous variable), b) the average daily activity time was divided into the quartiles called as the quartiles of MVPA duration.

Secondly, children were asked to evaluate how many times they were physically active in their weekly out-of-school-hours (times per week). 10 response options were possible, ranging from ‘I’m not physically active at all’ to ‘seven times or more per week’. For the analyses, two types of variables were formed: a) the continuous MVPA frequency based on the original answers, and b) the categorized MVPA frequency, which was recoded from the original variable so that answer options from ‘never’ to ‘1-3 times per month’ was coded to 0.5. The other answer options were coded from 1 (‘one time per week’) to 7 (‘seven time or more per week’).

### Accelerometer data management

MVPA was assessed by the Actigraph GTX3 (LLC, Florida, USA) accelerometer, an validated construct measure of MVPA [16]. The accelerometer was worn on the waist seven consecutive days except when in water. Actigraph data was analyzed by separating out-of-school-hours from the sleeping times and school times by Actilife 5.1. The epoch length was set at 15 seconds. Non-wear time was defined as 60 minutes of consecutive zeroes. We chose to use Evenson’s cut-points [17] which are recommended for use with school-aged children [18]. That is, the cut-point for at least moderate activity is 2296 counts per minute [17].

A valid day for the analysis was defined as at least eight hours of data in the child’s out-of-school-hours. Each child had to have four days of valid data with one weekend day. The total minutes of vigorous and moderate activity in out-of-school-hours indicated by the accelerometer were combined to form a moderate-to-vigorous-activity measure (MVPA). The average minutes of MVPA in children’s out-of-school-hours per day was calculated by dividing the total amount of MVPA in the selected four days by four.

### Analysis

All the analyses were conducted by using SPSS software, version 19.0 (SPSS, Chicago USA). To test the repeatability of questions, intra-class correlations (ICC) with 95 % confidence intervals were calculated using a two-way random model with an absolute agreement type [19].

To test the validity, the self-reported daily MVPA duration and frequency were compared to the accelerometer MVPA minutes and by calculating the Spearman’s correlations with 95 % confidence intervals [20]. Bland-Altman plots with 95 % limits of agreement were calculated to measure the agreement between and within average daily MVPA duration (min/day) according to the questionnaire and the average daily MVPA (min/day) according to the accelerometer.

The Kruskal-Wallis tests with pairwise comparisons using the Dunn-Bonferroni correction were done for testing if the questionnaire was able to categorize children according to their levels of MVPA. The objectively measured MVPA was compared separately to a) the quartiles of self-reported MVPA duration and b) to the categorized self-reported MVPA frequency.

## Results

Sample characteristics are presented in Table 1. In total, 171 children (60 % of invited children) were willing to participate in this sub-study. Of these children 16 were not in school, when the introduction on using the accelerometer was given. Therefore, a total of 155 children (93 girls) wore accelerometer. The average time of wearing the accelerometer per day in out-of-school-hours was 10.6 hours (variation between 8.5 –12.5 h).

**Table 1:** Sample characteristics of 11-year old participants in validation study

		N	Missing values	Median	Lower quartile – Upper quartile
Accelerometer MVPA^1^ (min/day)					
	MVPA^1^	126	29	43.00	34.32 – 56.25
Self-reported MVPA^1^ (min/day) in measuring time 1.					
	MVPA^1^ duration (min/day)	149	6	60.00	42.85 – 77.14
	MVPA^1^ frequency (0.5 – 7) ^2^	151	4	5	4 – 6
Self-reported MVPA^1^ (min/day) in measuring time 2.					
	MVPA^1^ duration (min/day)	153	2	60.00	42.86 – 77.14
	1^st^ quartile (8.00 – 42.86 min/day)	44		34.29	
	2^nd^ quartile (42.9 – 60.00 min/day)	47		51.43	
	3^rd^ quartile(60.00 – 77.14 min/day)	30		68.57	
	4^th^ quartile(77.15 – 86.00 min/day)	32		85.71	
	MVPA^1^ frequency (0.5 –7 ) ^2^	155	0	5	4 – 7

^1^ PA=Physical activity

^2^ Answer options from not at all to one to three times per week coded as 0.5, answer options from one time per week to seven times per week or more coded from 1 to 7.

The ICC of MVPA duration was .65 and MVPA frequency .64. The Spearman correlation between the accelerometer-measured MVPA and MVPA duration was .25. The Spearman correlation between the accelerometer-measured MVPA and MVPA frequency was .25.

The Bland-Altman plot of MVPA (plot not shown) shows a variation between the MVPA measured by accelerometer and MVPA duration measured by questionnaire. The mean difference between two measurement methods were - 6.3 minutes, but there was no proportional bias indicating the level of agreement (t-value -.648, p-value .52).

**Fig. 1:**
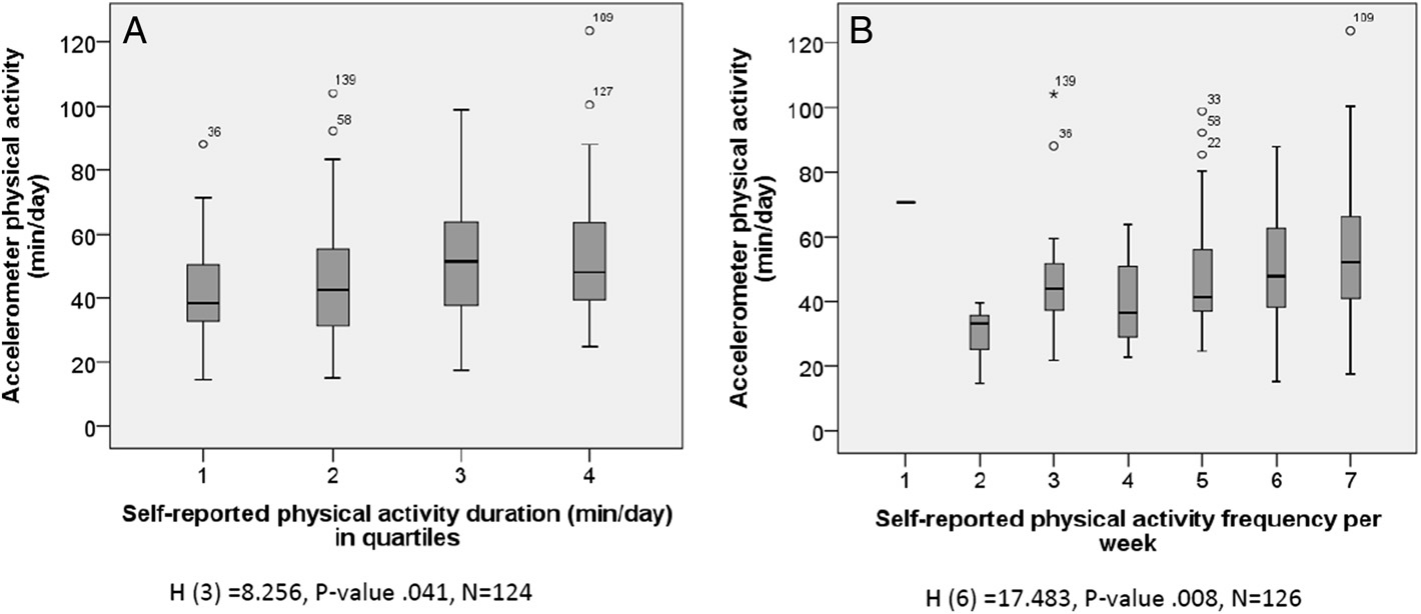
Validity of physical activity duration (**a**) and frequency (**b**) compared to accelerometer measurement. Legend: The box represents the median and interquartile range and the bars represent the range as measured by Kruskal Wallis test. Graphs are presented for accelerometer minutes (min/day). Only the data with observations are shown in Fig. 1

Fig. 1 shows the box-and-whisker plots describing the results of the Kruskal-Wallis tests. The lowest quartile of children according to the MVPA duration (Fig. 1a) question had less MVPA minutes according to accelerometer than the highest quartile of children (H(3) = 8.256, p-value .041). Pairwise comparisons found no significant differences between the quartiles. The children (Fig. 1b), who reported being less frequently physically active, had less MVPA minutes according to accelerometer than the children, who answered more frequent PA (H(6) = 17.483, p-value .008). Pairwise comparisons indicated that there was a significant difference between groups of 2 and 7 (difference = −49.32 minutes, adjusted p-value .020).

## Discussion

The present study identified that the repeatability of children’s out-of-school PA questionnaire was substantial [21], and the validity was poor [20]. The Bland-Altman plot of PA did not display any proportional bias indicating level of agreement between two measurements. The questionnaire categorized correctly the 11-year old children according to the objectively measured MVPA.

The repeatability of this questionnaire was similar to those from other studies that have been conducted in school-aged children [7, 8], but slightly better than in another questionnaire focusing on the out-of-school timeframe [22]. 11-year-old Finnish children’s PA activities in out-of-school-hours usually happen in regular trainings that are hold at similar frequency and duration after school-hours. Therefore, children can easily recall of their PA duration and frequency.

The validity correlation is in line with other validation studies focusing on out-of-school timeframe [7, 22], and lower than in many PA questionnaires that have not focused in certain timeframe [7, 22]. The explanation for poor correlation might be that the accelerometer measures the exact duration of activity in minutes whereas the questionnaire measures children’s comprehension of activities in hours. Thus, for each one hour 'training' or 'exercising,' the accelerometer measures the exact number of minutes in actual PA, whereas children report the entire hour (i.e. time in actual PA, breaks and rest periods combined). It might also be that one week timeframe was too long to recall. The questions could have been asked separately for weekdays and weekends to provide more accurate information. In addition, the accelerometer data was only collected over one week, and this measured week may not be a representative week, whereas the questionnaire measures the habitual activity. Future studies might need to include objective measurement in their protocols beside the self-report to avoid the miss-reporting.

The questionnaire had a moderate capability to categorize a group of children according to their MVPA levels. The children, who reported more MVPA frequency and duration, had more objectively measured MVPA. Based on this finding, the questionnaire is therefore able to discriminate between physically active and physically inactive, in out-of-school hours. This finding might be beneficial for future large-scale population level studies that might not be able to conduct objective measurements, but also for the health promotion practitioners, who can discriminate children’s activity levels with the help of two questions.

This study has some limitations. The difference between two measurement times of questionnaire was large, which might have had impact on the repeatability of questionnaire. In addition, the use of certain cut-off point is a possible cause for disagreement when compared with other PA estimates such as self-reports. However, the chosen cut-points are recommended to use for this age group [18]. Only two dimensions (frequency and duration) of PA were validated in this study. The weakness of this study was that questions about the type and the intensity of PA were not included in the questionnaire.

The strength of this study was that the repeatability and validity of the questionnaire was tested by conducting several analyses conducting both individual and group-level comparison between the measures. The second strength of this study was that it examined the validity and reliability of PA focusing in out-of-school timeframe. This timeframe in Finnish context is important, because the most of variation in children’s activities happen at that time. In addition, this questionnaire as consisting only of two questions does not require too much attention from children. Shortness of questionnaire benefits also future studies and health practitioners.

## Conclusions

The repeatability of the questionnaire is substantial, whereas the validity is poor. If the aim is to classify children according to their behavior, the questionnaire is moderately appropriate.

## References

[1] Biddle SJ, Gorely T, Stensel DJ (2004). Health-enhancing physical activity and sedentary behaviour in children and adolescents. J Sports Sci.

[2] Biro F, Wien M (2010). Childhood obesity and adult morbidities. Am J Clin Nutr.

[3] Corder K, van Sluijs EM, Ekelund U, Jones AP, Griffin SJ. Changes in children's physical activity over 12 months: longitudinal results from the SPEEDY study. Pediatrics. 2010;126(4):e926–35. 10.1542/peds.2010-0048 20837590

[4] .Currie C, Zanotti C, Morgan A, Currie D, de Looze M, Roberts Ch, et al. eds. Social determinants of health and well-being among young people. Health Behaviour in School-aged Children (HBSC) study: international report from the 2009/2010 survey. Copenhagen: WHO Regional Office for Europe; 2012.

[5] .Perusopetusasetus. (The Finnish Act of Basic Education). 1998/852. http://www.finlex.fi/fi/laki/alkup/1998/19980852. Accessed 4 Jan 2016.

[6] .Opetushallitus, Board of Education, Koulun kerhotoiminnan esite (The Brochure of activity in the Finnish School Clubs).2014. www.edu.fi/perusopetus/kerhotoiminta. Accessed 4 Jan 2016.

[7] Helmerhorst HJ, Brage S, Warren J, Besson H, Ekelund U (2012). A systematic review of reliability and objective criterion-related validity of physical activity questionnaires. Int J Behav Nutr Phys Act.

[8] Lubans DR, Hesketh K, Cliff DP, Barnett LM, Salmon J, Dollman J, et al. A systematic review of the validity and reliability of sedentary behaviour measures used with children and adolescents. Obes Rev. 2011;12(10):781–99. 10.1111/j.1467-789X.2011.00896.x 21676153

[9] Sallis JF (1991). Self-report measures of children's physical activity. J Sch Health.

[10] Sirard JR, Pate RR (2001). Physical activity assessment in children and adolescents. Sports Med.

[11] Kohl HW, Fulton JE, Caspersen CJ (2000). Assessment of Physical Activity among Children and Adolescents: A Review and Synthesis. Prev Med.

[12] Sallis JF, Saelens BE (2000). Assessment of physical activity by self-report: status, limitations, and future directions. Res Q Exerc Sport.

[13] Jago R, Baranowski T, Baranowski JC, Cullen KW, Thompson DI (2007). Social desirability is associated with some physical activity, psychosocial variables and sedentary behavior but not self-reported physical activity among adolescent males. Health Educ Res.

[14] Klesges LM, Baranowski T, Beech B, Cullen K, Murray DM, Rochon J, et al. Social desirability bias in self-reported dietary, physical activity and weight concerns measures in 8- to 10-year-old African-American girls: results from the Girls Health Enrichment Multisite Studies (GEMS). Prev Med. 2004;38(Suppl):S78–87. 10.1016/j.ypmed.2003.07.003 15072862

[15] Trost SG (2007). State of the Art Reviews: Measurement of Physical Activity in Children and Adolescents. Am J Lifestyle Med.

[16] Hanggi JM, Phillips LR, Rowlands AV (2013). Validation of the GT3X ActiGraph in children and comparison with the GT1M ActiGraph. J Sci Med Sport.

[17] Evenson KR, Catellier DJ, Gill K, Ondrak KS, McMurray RG (2008). Calibration of two objective measures of physical activity for children. J Sports Sci.

[18] Trost SG, Loprinzi PD, Moore R, Pfeiffer KA (2011). Comparison of accelerometer cut points for predicting activity intensity in youth. Med Sci Sports Exerc.

[19] Field A (2009). Discovering statistics using SPSS: 3rd ed.

[20] Streiner DI, Norman GR (2003). Health measurement scales: a practical guide to their development and use.

[21] Landis JR, Koch GG (1977). The measurement of observer agreement for categorical data. Biometrics.

[22] Telford A, Salmon J, Jolley D, Crawford D. Reliability and validity of physical activity questionnaires for children: the children's leisure activities study survey (CLASS). Ped Exerc Sci. 2004;16(1):64–78. 10.1123/pes.21.3.339 19827457

